# Editorial of Special Issue “The 2nd Edition: Vaccines for Aquaculture”

**DOI:** 10.3390/vaccines10081242

**Published:** 2022-08-03

**Authors:** Beatriz Novoa, Patricia Pereiro

**Affiliations:** Instituto de Investigaciones Marinas (IIM), Consejo Superior de Investigaciones Científicas (CSIC), C/Eduardo Cabello 6, 36208 Vigo, Spain

The Special Issue “Vaccines for Aquaculture” of the journal *Vaccines* had a great success among fish immunologists, with 17 published manuscripts. In this new edition, “The 2nd Edition: Vaccines for Aquaculture”, we would like to continue increasing and favoring the knowledge and advances in the development of fish vaccines.

Fish vaccination is routinely used in finfish aquaculture. Nevertheless, whereas vaccination is well established for some fish species, there is a lack of adequate vaccination strategies for a multitude of fish species and against certain pathogens causing economic losses in the aquaculture industry [1]. Moreover, to reduce the cost of the vaccines by implementing new production systems, minimize the manipulation of the animals (e.g., by administering mucosal vaccines or polyvalent preparations), and increase the efficiency of the vaccines through different approaches, such as the development of novel adjuvants, are some of the challenges that the fish immunologists need to face [2].

The evolution of fish vaccinology was especially fast for the most economically relevant fish species or those with a wider geographical distribution area. This is the case of European sea bass (*Dicentrarchus labrax*), Nile tilapia (*Oreochromis niloticus*), or Atlantic salmon (*Salmo salar*). Miccoli et al. [3] summarized and discussed in their review all the information available about the development of vaccines against viruses, bacteria and parasites in these three species. The authors highlighted the significant progress in fish vaccinology for these teleost species during the last decades, which is reflected by the high number of scientific publications in this regard, including information about attenuated, inactivated, subunit, recombinant, and DNA vaccines [3]. Putting together all that information allows for better identifying the strengths and weaknesses of the different vaccination approaches and provides helpful guidance for future decision making.

Inactivated pathogen vaccines are probably the most widely used strategies in fish vaccinology. Nevertheless, the inactivation procedure is pivotal for developing an adequate immune response in immunized fish. It is needed to achieve a compromise between efficient pathogen inactivation and retention of antigenicity. Three works were published in the previous Special Issue showing the efficiency of inactivated vaccines against viruses. Zeng et al. [4] reported that β-propiolactone inactivation of tilapia-lake virus (TiLV) showed higher protective efficacy in Nile tilapia than that conducted with formalin, probably the most widely used inactivating compound. Moreover, the β-propiolactone-inactivated viral particles showed a great ability to produce neutralizing antibodies against TiLV, reduce the viral loads in different tissues from tilapia and induce a strong immune response even after 6 weeks post primary immunization [4]. Nervous necrosis virus (NNV) has been attracting a lot of attention over the last few years due to the wide spectrum of species it affects and the great economic impact it generates in the aquaculture sector. Valero et al. [5] designed a binary ethylenimine (BEI)-inactivated vaccine against NNV and compared its efficacy with a formalin-inactivated vaccine in Senegalese sole (*Solea senegalensis*). The results revealed similar protection for both types of vaccines, but, interestingly, the production of IgM-NNV antibodies was only induced by the BEI-inactivated vaccine, and a different array of immune genes was induced by both vaccines [5]. These results could indicate that different inactivation methods would elicit different immune mechanisms in the immunized fish, although, in both cases, the vaccines reduced the mortality caused by NNV [5]. These observations open the door for further investigations based on the understanding of these differential responses. Finally, Falco et al. [6] evaluated three inactivation procedures for NNV (heat-, UV-, and formalin-inactivated methods). Their investigation concluded that NNV particles were highly resistant to heat inactivation, whereas the UV-irradiation and formalin treatments showed promising results as NNV-inactivation procedures [6]. Nevertheless, as it was mentioned above, maintaining the antigenicity of the pathogen is pivotal for the efficiency of the inactivated vaccines. To determine the antigen quality of inactivated infectious spleen and kidney necrosis virus (ISKNV) particles, Liang et al. [7] designed a specific double-antibody sandwich ELISA for detecting the major capsid protein (MCP) antigen. This test would allow to quantitatively determine the concentration of MCP antigen, which would be also a quality indicator of the inactivation method [7].

Attenuated vaccines are also a frequent strategy in vaccinology. However, in some cases, the mutations resulting in pathogen attenuation remain unexplored. This information is necessary in order to better understand the mechanisms involved in the attenuation and the safety of these vaccines. Cai and Arias [8] sequenced the genomes of the attenuated mutant used as a vaccine against the bacterial pathogen *Flavobacterium columnare* (Fc1723) and the parent virulent strain (FcB27). Interestingly, the authors found 16 single nucleotide polymorphisms (SNPs), some of them located on genes involved in bacterial attachment and extracellular protease secretion activity, two mechanisms contributing to attenuated virulence [8]. They also detected mutations in multiple genetic loci that contribute to its stability, which could indicate that the attenuated strain would revert to its virulent form very unlikely [8].

The ideal administration methods for vaccines are those requiring low fish handling and not limited by the size of the individuals. Because of this, the development of mucosal vaccines for oral or immersion administration is experiencing extraordinary development. Moreover, mucosal vaccines usually elicit higher protective responses at the mucosal surfaces, which are the main portals of entry for most of the pathogenic microorganisms [9]. However, these administration routes have to face some problems since antigen uptake is low compared to injected vaccines and a high quantity of antigens is needed to achieve positive results. Moreover, orally administered vaccines are quickly degraded due to the low pH of the stomach. To favor the internalization of the vaccines and reduce their degradation, different vaccine delivery systems, known as nanocarriers, have been explored. Kitiyodom et al. [10] tested the immune response elicited in red tilapia by the administration of a formalin-inactivated vaccine against *F. columnare* encapsulated in a mucoadhesive polymer chitosan-complexed nanovaccine through immersion. The vaccination procedure induced higher protection against a *F. columnare* challenge, stronger production of specific antibodies, and powerful modulation of immune-related genes compared to the normal formalin-killed vaccine [10]. Polyhedra produced by *Bombix mori* cytoplasmic polyhedrosis virus (BmCPV) are also an attractive nanocarrier. These polyhedra are protein crystals produced by the virus in the cytoplasm of infected cells and contain a multitude of viral particles for protecting the embedded viral particles. By fusing a specific tag sequence from the polyhedrin gene to sequences encoding for foreign proteins, recombinant proteins encapsulated in these polyhedral can be obtained. This strategy was followed by Zhang et al. [11] to design two encapsulated vaccines against cyprinid herpesvirus II (CyHV-2). Both vaccines, containing two different antigens, increased the survival rate after a challenge with CyHV-2 in gibel carp (*Carassius auratus* gibelio) [11]. Although the vaccines were administered by intraperitoneal (i.p.) injection, these results suggest that this technology could be very useful for mucosal vaccination, as they are also highly stable at room temperature [11].

Interestingly, extracellular membrane vesicles (MVs) secreted by bacteria, which contain different proteins, DNA, RNA, and virulence factors, could also serve as naturally encapsulated multi-antigen vaccines. Mertes et al. [12] purified MVs from two *Francisella* species and tested by i.p. injection their protective and immunomodulatory effects in Nile tilapia and Atlantic cod (*Gadus morhua).* Contrary to previous reports on fish, these authors did not observe significant protection and/or immune modulation after MVs administration compared to the control groups, which could indicate that the immunization mediated by these particles is not useful against Francisellosis and/or in certain fish species [12].

Another way to encapsulate vaccines is to do it into the live feed, such as *Artemia salina* or rotifers. Dang et al. [13] encapsulated a *Vibrio anguillarum* bacterin in *A. salina* nauplii and administered this bio-encapsulated vaccine to lumpfish (*Cyclopterus lumpus*) larvae. Modulation of certain immune genes, including interleukins, chemokines, and different immunoglobulin genes, among others, was observed at 2 and 4 weeks post-immunization [13]. After 9 months, the fish were orally boosted with the bio-encapsulated vaccine or by i.p. injection of the bacterin; two months after boost immunization, fish were infected with *V. anguillarum*, and only those receiving the i.p. boost showed a significantly higher survival [13]. Based on this, the authors concluded that, although the administration of the oral bio-encapsulated vaccine induced a certain immune modulation, this vaccine probably did not have the ability to cross the endothelium and reach deep lymphoid tissues to trigger immune protection [13].

The development of vaccines based on the administration of bacterial biofilms is an innovative technique that is gaining popularity in recent years. Bacteria growing in multicellular communities (biofilms) produce an extracellular matrix with a multitude of exoproteins that could serve as attractive antigens for immunization [14]. These exoproteins are not produced when the bacteria are grown under planktonic conditions, which could limit the protection against those pathogenic bacteria forming biofilms [14]. Bacterial biofilms can be produced when bacteria grow in a nutrient-depleted media and with an appropriate substrate for their attachment. Su and Chen [15] developed an oral biofilm vaccine against *Photobacterium damselae* subsp. *damselae* and tested its efficacy in giant grouper (*Epinephelus lanceolatus*). *P. damselae* biofilms were allowed to grow on chitosan particles, and then they were inactivated with formalin to be incorporated into the fish diet, which was provided daily for a period of two weeks. After vaccination, fish returned to their basal diet for two additional weeks before the immersion challenge with the pathogenic bacteria [15]. The biofilm vaccine significantly reduced the mortality caused by the bacteria, whereas the whole-cell vaccine did not induce any significant protection; moreover, blood leukocyte phagocytosis, the level of specific antibodies in serum, and the modulation of different pro-inflammatory genes in the spleen were significantly higher in the animals receiving the biofilm vaccines compared to the controls and the fish receiving the whole-cell vaccine [15]. These results provide interesting clues for further applications of these vaccines in aquaculture.

Developing efficient polyvalent vaccines would provide numerous advantages to the fish farmers since fish would be immunized against several pathogens with only one vaccine. Nevertheless, the optimization of this type of vaccine could not be easy since optimal preservatives for some antigens could not be suitable for others, multiple antigens can result in an exacerbated reactogenicity, or a reduced titer of antibodies could be achieved for the different pathogens due to the presence of immunosuppressive epitopes and antigen competition or interference, among others [16]. One ideal solution for these associated problems is to identify common conserved antigens from different pathogens to generate recombinant vaccines. Based on this, Chukwu-Osazuwa et al. [17] screened the proteome of five of the most frequent pathogens in finfish aquaculture (*Piscirickettsia salmonis*, *Aeromonas salmonicida*, *Yersinia ruckeri*, *Vibrio anguillarum,* and *Moritella viscosa*) and identified unique and common antigens for these species. Further analyses of common outer membrane antigens revealed a relatively low sequence identity but good structural homology, and potential B cell and T cell epitopes from these common antigens were identified and docked to Atlantic salmon and lumpfish MHC class II [17]. A total of 13 epitopes belonging to five common antigens could interact with CD4+ T and B cells and could serve as a base for developing an anti-bacterial polyvalent vaccine [17].

As it was mentioned above, bivalent or polyvalent vaccines can result in decreased immunogenicity due to cross-reactions among different antigens. Therefore, it is important to know how the animals respond to this type of vaccine. Lim and Hong [18] evaluated the transcriptome response in the head kidney of rainbow trout (*Oncorhynchus mykiss*) i.p. injected with a bivalent formalin-inactivated vaccine against *A. salmonicida* and *V. anguillarum*. The vaccine significantly induced the expression of gene markers of an activated immune response, such as TCRα, T-bet, sIgM, and mIgM, but RNA-Seq analyses also revealed the modulation of a multitude of other immune-related genes at 1, 3, and 5 days post-immunization. These results provide interesting information for understanding the response to this combined vaccine and lays the foundation for improving vaccine formulations [18]. Indeed, knowing the immune processes elicited by vaccines and their impact on the clinical signs induced by pathogens is especially interesting for those diseases not causing severe mortality episodes. This is the case of the salmon pancreatic disease virus (SPDv), which induces loss of appetite, lethargy and histological damage in different tissues of Atlantic salmon, but the impact of the disease can be reduced by minimizing the stress and controlling feeding. Collins et al. [19] designed a DNA vaccine against SPDv and conducted a kinetic analysis of the immune modulation induced by the vaccine, but also its ability to suppress viremia by eliminating the virus and preventing disease pathology. Moreover, the results provide interesting data about the infection kinetic pattern, even though some of them are useful as non-destructive methods (e.g., immune analyses in blood) that would allow monitoring the disease progression [19].

Adjuvants are substances that, combined with a specific antigen, produce a more robust immune response than the antigen alone through different mechanisms [20]. Nevertheless, certain adjuvants can cause local tissue damage and necrosis at the injection site [20]. Therefore, using an adequate adjuvant is pivotal for promoting an optimal immune response without inducing severe damage. Torres-Corral et al. [21] evaluated the efficacy of a bivalent vaccine against *V. anguillarum* and *A. salmonicida* subsp. *achromogenes* in turbot (*Scophthalmus maximus*) adjuvated with the non-mineral oil Montanide^TM^ and compared its effect to that induced by a commercial vaccine against these pathogens, which was adjuvated in liquid paraffine (AlphaJect 3000, Pharmaq AS). Moreover, they also tested the effect of Montanide^TM^ and the liquid paraffin Eolane 130. Although the autogenous vaccine induced long-lasting protection against both pathogens, these fish showed the highest degree of the impaired physiological parameter (damage in the peritoneal cavity, lower fish weight, hepatosomatic index, and higher viscerosomatic index, among others); however, these side effects could be associated with the use of Montanide^TM^ as adjuvant since the administration of this adjuvant alone also impacted some physiological parameters [21]. On the contrary, liquid paraffine Eolane 130 seemed to be well-tolerated by the animals and could serve as a promising candidate for future vaccine development [21].

Another interesting way to stimulate the immune response and potentiate the effect of the vaccines is to use the synthetic double-stranded RNA molecule Poly I:C as adjuvant. Chun et al. [22] found that Poly I:C potentiated the immune effects of a formalin-inactivated viral hemorrhagic septicemia virus (VHSV) vaccine in olive flounder (*Paralichthys olivaceus*). Higher levels of CD3+, CD4-1+ and CD4-2+ T cells were observed in most of the tested tissues and sampling points with the vaccine supplemented with Poly I:C compared to the vaccine alone, revealing the potential of this immunostimulant for its introduction in the vaccination protocols against fish viruses [22].

Finally, another challenge for the fish vaccine producers is to easily obtain a large-scale production of antigens. A strategy proposed by Luo et al. [23] for viral vaccine production in a stirred bioreactor consisted of the use of microcarriers and suspension culture systems of the host cells to maximize the obtention of viral particles since the attachment surface of these microcarriers is higher compared to the conventional monolayer culture. They optimized and evaluated the attachment and proliferation of Chinese perch brain (CPB) cells to the commercial microcarrier Cytodex 1 (GE HealthCare) to produce ISKNV and *Siniperca chuatsi* rhabdovirus (SCRV), obtaining very positive results. The study provided the optimal technical parameters for scale production of CPB cells for ISKNV and SCRV vaccine production [23].

Overall, these 17 contributions published in the Special Issue “Vaccines for Aquaculture” demonstrated that the research in this field is still in progress and that different aspects of the finfish vaccination are subjected to investigation. Although the information provided by these manuscripts will be very useful for the development of fish vaccines, there is still a long way to go. We hope that this new Special Issue, “The 2nd Edition: Vaccines for Aquaculture”, will serve as a platform to continue favoring the diffusion of the main findings in this field.

## References

[1] Adams A. (2019). Progress, Challenges and Opportunities in Fish Vaccine Development. Fish Shellfish Immunol..

[2] Mondal H., Thomas J. A Review on the Recent Advances and Application of Vaccines against Fish Pathogens in Aquaculture. Aquac. Int..

[3] Miccoli A., Manni M., Picchietti S., Scapigliati G. (2021). State-of-the-Art Vaccine Research for Aquaculture Use: The Case of Three Economically Relevant Fish Species. Vaccines.

[4] Zeng W., Wang Y., Hu H., Wang Q., Bergmann S.M., Wang Y., Li B., Lv Y., Li H., Yin J. (2021). Cell Culture-Derived Tilapia Lake Virus-Inactivated Vaccine Containing Montanide Adjuvant Provides High Protection against Viral Challenge for Tilapia. Vaccines.

[5] Valero Y., Olveira J.G., López-Vázquez C., Dopazo C.P., Bandín I. (2021). BEI Inactivated Vaccine Induces Innate and Adaptive Responses and Elicits Partial Protection upon Reassortant Betanodavirus Infection in Senegalese Sole. Vaccines.

[6] Falco A., Bello-Perez M., Díaz-Puertas R., Mold M., Adamek M. (2021). Update on the Inactivation Procedures for the Vaccine Development Prospects of a New Highly Virulent RGNNV Isolate. Vaccines.

[7] Liang H., Zhang L., Fu X., Lin Q., Liu L., Niu Y., Luo X., Huang Z., Li N. (2021). Development of a Double-Antibody Sandwich ELISA for Rapid Detection of the MCP Antigen Concentration in Inactivated ISKNV Vaccines. Vaccines.

[8] Cai W., Arias C.R. (2021). Deciphering the Molecular Basis for Attenuation of *Flavobacterium columnare* Strain Fc1723 Used as Modified Live Vaccine against Columnaris Disease. Vaccines.

[9] Muñoz-Atienza E., Díaz-Rosales P., Tafalla C. (2021). Systemic and Mucosal B and T Cell Responses Upon Mucosal Vaccination of Teleost Fish. Front. Immunol..

[10] Kitiyodom S., Yata T., Thompson K.D., Costa J., Elumalai P., Katagiri T., Temisak S., Namdee K., Rodkhum C., Pirarat N. (2021). Immersion Vaccination by a Biomimetic-Mucoadhesive Nanovaccine Induces Humoral Immune Response of Red Tilapia (*Oreochromis* sp.) against *Flavobacterium columnare* Challenge. Vaccines.

[11] Zhang T., Gu Y., Liu X., Yuan R., Zhou Y., Dai Y., Fang P., Feng Y., Cao G., Chen H. (2021). Incidence of *Carassius auratus* Gibelio Gill Hemorrhagic Disease Caused by CyHV-2 Infection Can Be Reduced by Vaccination with Polyhedra Incorporating Antigens. Vaccines.

[12] Mertes V., Bekkelund A.K., Lagos L., Ciani E., Colquhoun D., Haslene-Hox H., Sletta H., Sørum H., Winther-Larsen H.C. (2021). The Use of Extracellular Membrane Vesicles for Immunization against Francisellosis in Nile Tilapia (*Oreochromis niloticus*) and Atlantic Cod (*Gadus morhua* L.). Vaccines.

[13] Dang M., Cao T., Vasquez I., Hossain A., Gnanagobal H., Kumar S., Hall J.R., Monk J., Boyce D., Westcott J. (2021). Oral Immunization of Larvae and Juvenile of Lumpfish (*Cyclopterus lumpus*) against *Vibrio anguillarum* Does Not Influence Systemic Immunity. Vaccines.

[14] Loera-Muro A., Guerrero-Barrera A., Tremblay D.N.Y., Hathroubi S., Angulo C. (2021). Bacterial Biofilm-Derived Antigens: A New Strategy for Vaccine Development against Infectious Diseases. Expert Rev. Vaccines.

[15] Su F.-J., Chen M.-M. (2022). Protective Efficacy of Novel Oral Biofilm Vaccines against *Photobacterium damselae* subsp. *damselae* Infection in Giant Grouper, *Epinephelus lanceolatus*. Vaccines.

[16] Schlingmann B., Castiglia K.R., Stobart C.C., Moore M.L. (2018). Polyvalent vaccines: High-maintenance heroes. PLoS Pathog..

[17] Chukwu-Osazuwa J., Cao T., Vasquez I., Gnanagobal H., Hossain A., Machimbirike V.I., Santander J. (2022). Comparative Reverse Vaccinology of *Piscirickettsia salmonis*, *Aeromonas salmonicida*, *Yersinia ruckeri*, *Vibrio anguillarum* and *Moritella viscosa*, Frequent Pathogens of Atlantic Salmon and Lumpfish Aquaculture. Vaccines.

[18] Lim J., Hong S. (2021). Transcriptome Analysis in the Head Kidney of Rainbow Trout (*Oncorhynchus mykiss*) Immunized with a Combined Vaccine of Formalin-Inactivated *Aeromonas salmonicida* and *Vibrio anguillarum*. Vaccines.

[19] Collins C., Lester K., Del-Pozo J., Collet B. (2021). Non-Lethal Sequential Individual Monitoring of Viremia in Relation to DNA Vaccination in Fish–Example Using a Salmon Alphavirus DNA Vaccine in Atlantic Salmon *Salmo salar*. Vaccines.

[20] Awate S., Babiuk L.A., Mutwiri G. (2013). Mechanisms of Action of Adjuvants. Front. Immunol..

[21] Torres-Corral Y., Girons A., González-Barreiro O., Seoane R., Riaza A., Santos Y. (2021). Effect of Bivalent Vaccines against *Vibrio anguillarum* and *Aeromonas salmonicida* Subspecie *achromogenes* on Health and Survival of Turbot. Vaccines.

[22] Chun J.H., Jung J.W., Kim Y.R., Lazarte J.M.S., Kim S.W., Kim J., Thompson K.D., Kim H.J., Jung T.S. (2021). Poly (I:C)-Potentiated Vaccination Enhances T Cell Response in Olive Flounder (*Paralichthys olivaceus*) Providing Protection against Viral Hemorrhagic Septicemia Virus (VHSV). Vaccines.

[23] Luo X., Niu Y., Fu X., Lin Q., Liang H., Liu L., Li N. (2021). Large-Scale Microcarrier Culture of Chinese Perch Brain Cell for Viral Vaccine Production in a Stirred Bioreactor. Vaccines.

